# Supplementary material for: Encapsulated Bdellovibrio Powder as a Potential Bio-Disinfectant against Whiteleg Shrimp-Pathogenic Vibrios
Source: Microorganisms. 2019 Aug 7;7(8):244. doi: 10.3390/microorganisms7080244 (PMC6722716; doi:10.3390/microorganisms7080244)
Supplement: Supplementary file 1 [file microorganisms-07-00244-s001.pdf]

microorganisms

Supplementary Materials

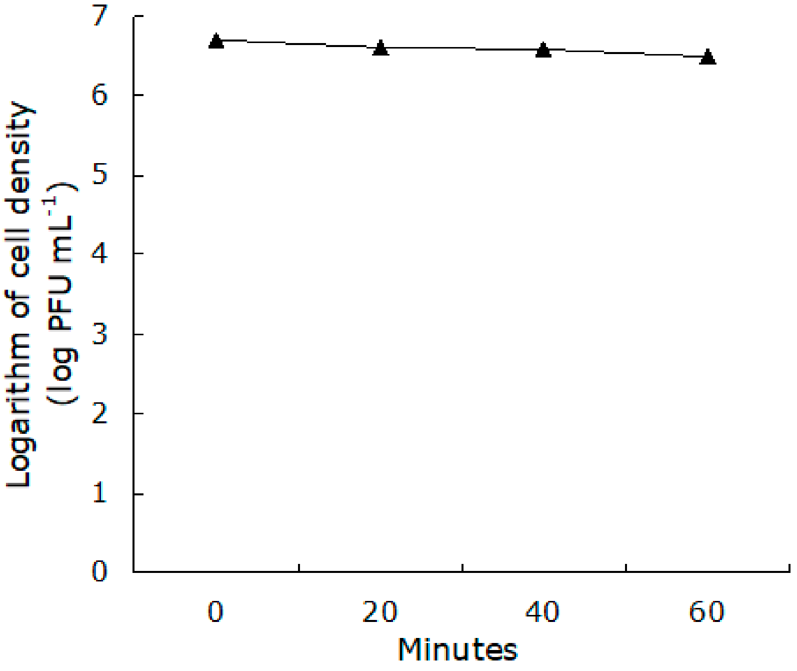

Figure S1. Survival of *Bdellovibrio* sp. strain F16 in a water bath at 50 °C.

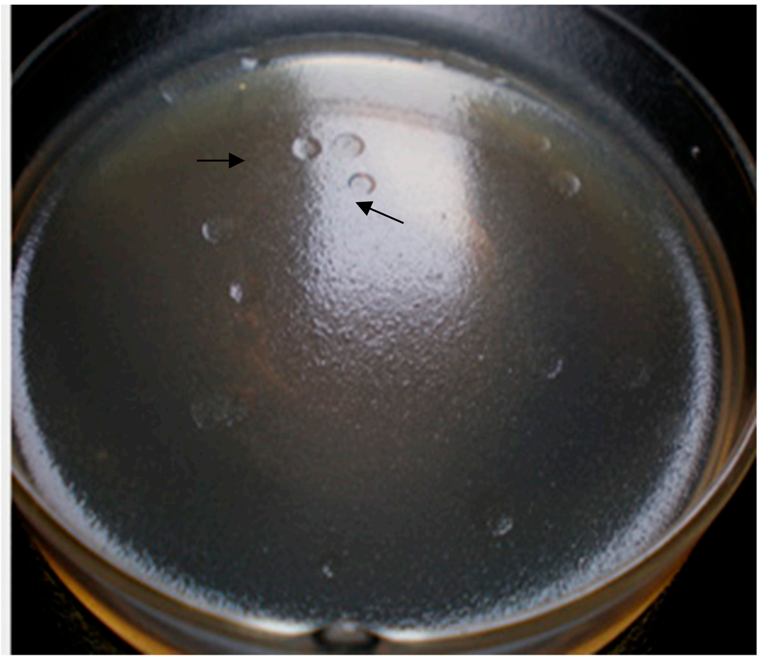

Figure S2. Plaques of *Bdellovibrio* sp. strain F16. Arrows show the round plaques formed on the double-layer agar plate after incubation for 48 h.
